# Oral Vaccine Delivery for Intestinal Immunity—Biological Basis, Barriers, Delivery System, and M Cell Targeting

**DOI:** 10.3390/polym10090948

**Published:** 2018-08-27

**Authors:** Sung Hun Kang, Seok Jin Hong, Yong-Kyu Lee, Sungpil Cho

**Affiliations:** 1 Department of Medical Sciences, College of Medicine, Hallym University, Chuncheon 24252, Korea; D18024@hallym.ac.kr; 2 Department of Otorhinolaryngology-Head and Neck Surgery, Hallym University, Dongtan Sacred Heart Hospital, Hwaseong 18450, Korea; enthsj@hallym.or.kr; 3 Department of Chemical and Biological Engineering, Korea National University of Transportation, Chungju 27469, Korea; 4 4D Biomaterials Center, Korea National University of Transportation, Jeungpyeong 27909, Korea

**Keywords:** oral vaccine delivery, M-cell targeting, intestinal immunity

## Abstract

Most currently available commercial vaccines are delivered by systemic injection. However, needle-free oral vaccine delivery is currently of great interest for several reasons, including the ability to elicit mucosal immune responses, ease of administration, and the relatively improved safety. This review summarizes the biological basis, various physiological and immunological barriers, current delivery systems with delivery criteria, and suggestions for strategies to enhance the delivery of oral vaccines. In oral vaccine delivery, basic requirements are the protection of antigens from the GI environment, targeting of M cells and activation of the innate immune response. Approaches to address these requirements aim to provide new vaccines and delivery systems that mimic the pathogen’s properties, which are capable of eliciting a protective mucosal immune response and a systemic immune response and that make an impact on current oral vaccine development.

## 1. Introduction

The immune system is the body’s natural defense against the entry or spread of pathogens. Within the immune compartments, the two most important systems are the peripheral lymphoid system and the mucosal immune system, which is commonly described by the acronym MALT (mucosa-associated lymphoid tissue) system [1]. The MALT system is comprised of various microcompartments containing lymphoid cells in epithelia (e.g., B cells, T cells, and accessory cells) and the lamina propria below mucosal sites, and is the principal mucosal inductive location where immune responses are initiated [2]. MALT system is involved in the predominant production of sIgA (secretory IgA) for mucosal immunity compared to the IgG-mediated systemic immunity. Therefore, the MALT system has been considered a critical target for the development of mucosal vaccines for preclinical and clinical trials [3,4,5,6].

The compartmentalization of the mucosal immune system restricts the choice of vaccination routes to induce immune responses at the desired effector sites [2]. Immunization with the cholera toxin B (CTB) subunit has revealed differential mucosal sIgA expression to be strongest at directly vaccine-exposed mucosa followed by adjacent mucosa or the interconnected compartments within the mucosal immune system [7]. Genital and rectal routes in humans have shown modest and localized immune responses at the inductive sites [7,8,9]. However, oral and nasal administration of CTB induces immune responses not only at the exposed site, but also in other mucosal compartments through the dissemination of sensitized lymphoid cells from the induction sites to the remote effector sites, such as the gut-mammary gland of lactating women or the cervicovaginal mucosa [7,9]. Therefore, the oral and nasal routes appear to be the most effective for advancing a mucosal vaccine for human use.

Nasal delivery of vaccines allows for the convenience of administration and has the potential for inducing both mucosal and systemic immune responses [6]. Licensed intranasal vaccines for humans include the influenza vaccines, FluMist^®^ Quadrivalent (intranasal spray; MedImmune, Gaithersburg, MD, USA) and the Nasovac-S™ (intranasal spray; Cipla, Mumbai, India) [10,11]. In addition, several nasal delivery products are at various stages of development in the preclinical and clinical pipelines (Table 1). Of these trials, the study of an intranasal HIV vaccine with a nanosphere delivery system loaded with HIV antigens demonstrated a successful immunization effect through elevation of immunoglobulin [12]. However, nasal administration of HIV antigen was recently terminated due to safety concerns. The concerns are side effects including weakness or paralysis on one side of facial muscles (e.g., Bell’s palsy) and damage to the olfactory nerves and nasal tissues. An alternative solution that avoids such side effects, oral vaccination garnered the attention of many scientists due to the physiological role of the gastrointestinal (GI) tract as the first line of defense against the mucosal entry of pathogens.

The intestine contains 70–80% of all immunoglobulin-producing cells in the body and predominantly produces sIgA, which is responsible for intestinal immunity [13]. The resistance of sIgA to intestinal proteases makes this immunoglobulin uniquely suited to be the first line of protection against invasive pathogens. Oral vaccination aims to induce intestinal immunity associated with the production of sIgA, which is difficult to achieve by systemic immunization through parenteral vaccination. Oral vaccination with appropriate adjuvants can induce both systemic and intestinal immunity. It has advantages in preventing infectious disease, as well as the colonization of pathogens at the intestinal surface [1,2]. In addition, oral vaccines utilize needle-free injection, which has contributed to the patient compliance as well as relative safety compared to parenteral vaccines. Therefore, it could be the best means of meeting the needs of populations having limited access to trained medical professionals [4]. Despite oral vaccines having these attractive features, the limited numbers of approved oral vaccines (Table 1) exemplify specific challenges in designing these vaccines. Ideal oral vaccines require to meet several criteria such as sufficient protection of antigens against gastrointestinal (GI) environment, high antigen loading or encapsulating capacity of vehicles, prolonged exposure of antigens to antigen-presenting cells, sufficient targeting ability to intestinal cells, especially intestinal microfold (M)-cells, enough production of long-term mucosal and systemic effect, and adequate safety. Therefore, the development of better oral vaccines is necessary to resolve issues with the enhancement of immune reactions by overcoming immunological and physiological barriers as well as oral tolerance [14,15,16]. In this article, we reviewed the biological basis of intestinal immunity, barriers to oral vaccine delivery, current progress in oral vaccine delivery systems, and possible directions to improve oral vaccination.

## 2. Biological Basis of Intestinal Immunity

**Figure 1:**
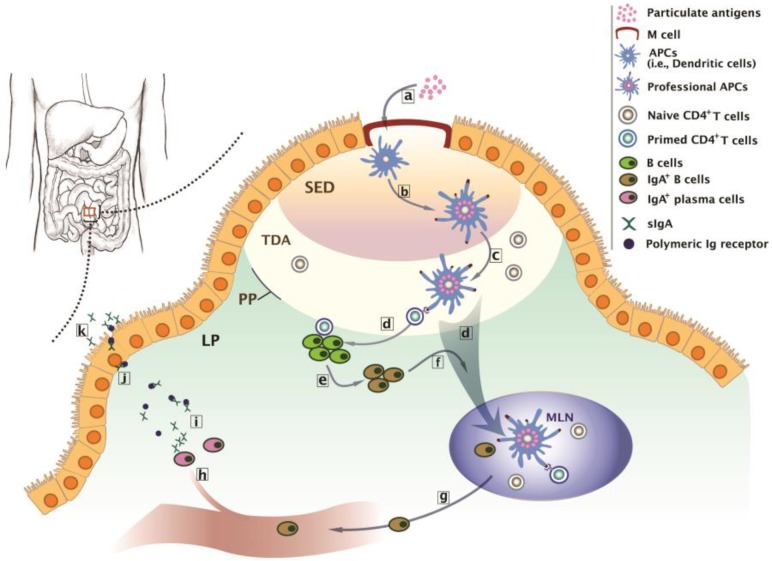
Biological basis of intestinal immunity at the Peyer’s patches (PP). (**a**) Transcytosis of particulate antigens to antigen presenting cells (APCs) such as DCs through M cell portal at the inductive sites. (**b**) Transformation of APCs to professional APCs after antigen-presentation at the subepithelial dome (SED). (**c**) Priming of naïve CD4^+^ T cells by professional APCs at the thymus-dependent area (TDA). (**d**) Activation of B cells by the primed CD4^+^ T cells, or active migration of professional APCs to the mesenteric lymph node (MLN) for further CD4^+^ T cell activation and subsequent IgA^+^ B cell production. (**e**) Transformation of B cells to IgA^+^ B cells. (**f**) Migration of IgA^+^ B cells to the MLN. (**g**) The entrance of IgA^+^ B cells to the systemic circulation through efferent lymph and thoracic ducts. (**h**) Accumulation of IgA^+^ B cells at the lamina propria (LP) and maturation of IgA^+^ B cells to IgA^+^ plasma cells. (**i**) The release of dimeric or polymeric IgA from the IgA^+^ plasma cells. (**j**) Migration of the complex of dimeric or polymeric IgA with polymeric Ig receptor toward the luminal surface of the intestine. (**k**) Transcytosis of the complex of and release sIgA at the effector sites.

Intestinal Peyer’s patches (PP) are dome-like, multicellular structures located in the intestine. PP are at their highest density in the human ileum and are comprised of 10–1000 individual follicles covered by a follicle-associated epithelium (FAE), a single layer of columnar epithelial cells [33]. Individual PP are an example of the gut-associated lymphoid tissue (GALT), a part of the MALT system. The biological basis of oral vaccination is the understanding of intestinal immunity triggered by antigens coming in contact with the intestinal inductive site. The intestinal inductive site is an organized tissue structure consisting of PP, mesenteric lymph nodes (MLN), and immunocytes including lymphocytes and accessory cells, such as dendritic cells (DCs) (Figure 1) [34]. Intestinal immunity starts from contact of an antigen with M cells presented in the FAE of PP. M cells have distinguishing characteristics such as the absence of microvilli, a thin glycocalyx on the apical surface, and the presence of an invaginated basolateral pocket which houses lymphocytes. These unique properties of M cells allow the translocation of luminal materials to the subepithelial compartment. Therefore, M cells are believed to be an intestinal portal for antigens, as well as invasive pathogens, such as *Salmonella*, *Shigella*, *Yersinia*, and reoviruses [34]. The antigen passing through the M cells meets antigen presenting cells (APCs), such as DCs. After uptake, processing, and presenting the antigen in the major histocompatibility complex II (MHC II). APCs become professional APCs and are involved in the immunologic priming of localized T cells. The primed T cells further activate the localized B cells, which have experienced immunoglobulin class switching and are becoming to IgA^+^ B cells. Some professional APCs and IgA^+^ B cells are actively transported to the MLN through draining lymphatic system. Naïve CD4^+^ T cells presented in the MLN encounter them and become antigen responsive CD4^+^ T cells, with the expression of α4β7 integrin and CCR9 chemokine receptors with homing affinity to the mucosa, the effector site. The antigen responsive CD4^+^ T cells leave the MLN through efferent lymph, enter the systemic circulation through the thoracic duct, and finally accumulate in the lamina propria of the mucosa. By massive production of cytokines, particularly interferon-γ (INF-γ) and interleukin-4 and -10 (IL-4 and -10), these antigen-experienced CD4^+^ T cells play the role of true immune effector cells by helping local B cells to become IgA^+^ plasma cells, memory cells for specific antigens, and regulatory cells responsible for maintaining local tolerance to environmental antigens [34,35,36]. This intestinal immunity suggests the possible biological basis of why oral vaccination is required to protect against intestinal infections, whereas parental vaccines are generally ineffective.

## 3. Barriers to Oral Vaccine Delivery

### 3.1. Physiological Barriers

**Figure 2:**
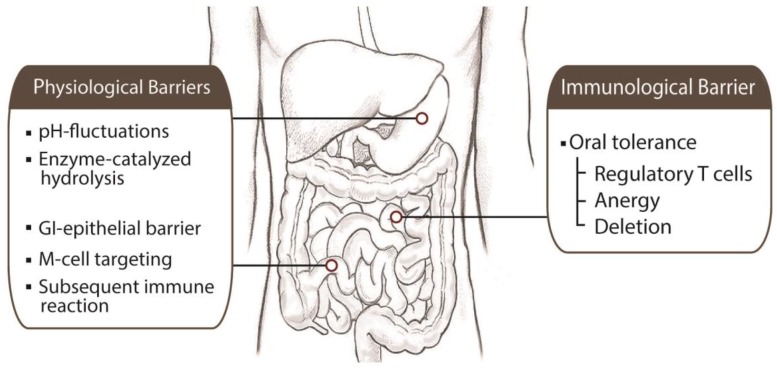
Physiological and immunological barriers to oral vaccine delivery. The different regions of the GI tract present physiological barriers to prevent the entrance of oral vaccine delivery vehicles onto the body. Once oral vaccines have reached the stomach and entered the small intestine, various conditions need to be overcome to achieve effective immunization. In addition to physiological barriers, oral tolerance plays an immunological barrier in the loss of desired immune response by induction of regulatory T cells and anergy or deletion of specific T cells.

Several gastrointestinal (GI) specific barriers contribute to the instability and lack of bioavailability of oral vaccines [15,16,37] (Figure 2). Enzyme-catalyzed hydrolysis and pH-fluctuations in the GI tract affect the stability of oral vaccines. They lead to the irreversible conformational changes and degradation of the antigens in oral vaccines. Moreover, the denatured vaccines lead to precipitation in the GI-lumen, loss of intestinal binding, and presentation of inactive epitopes to PP, which could induce a false immune response [16]. The GI-transit time is also a factor that influences the GI stability of the oral vaccine. Considering different gastric-emptying times, which can be as short as 2 h under fasting conditions or as long as 16 h under fed conditions, the prolonged presence of the vaccine in the stomach could result in proteolytic digestion or denaturation under the extremely low stomach pH (pH 2–4) [38].

Intestine-specific factors determine the bioavailability of oral vaccines. These factors are (i) the intrinsic protective role of GI epithelium [39], (ii) low numbers of M cells [2,33], (iii) M cell-specific targets [2], and (iv) induction of subsequent immune reaction [40,41]. Within the FAE of PP in the GI layer, enterocytes, goblet cells (GCs), and M cells comprise subsets [39]. The enterocytes are the most abundant GI cell types and block access of biologics (e.g., pathogens, vaccines) from the intestinal lumen to the interstitial spaces between cells by maintaining tight junctions. In addition, enterocytes secrete several defense enzymes such as lysozyme and phospholipase A2, which can hydrolyze protein and lipid components. Goblet cells (GCs) make up a relatively low percentage in the intestine compared to the enterocytes. However, GCs generate the intestinal mucus layer, which is a significant physical barrier in the delivery of vaccines to the intestinal target [42]. As mentioned previously, M cells are a portal of entry for pathogens and particles. Therefore, they represent potential targets to increase the effectiveness of oral vaccines. However, their low numbers in the PP of rodents (~10%) and humans (~5%) impacts the ability to achieve significant vaccine targeting [43,44]. Meynell et al. reported an increase in M cell numbers by exposure to *Streptococcus pneumonia* R36a [45]. With possible side effects, including food allergies and inflammatory disease, attention shifted to the development of improved targeting of existing M cells [2]. Utilization of M cell specific moieties is useful tools for targeting vaccines to M cells. Several ligands targeting M cell surface receptors, including Toll-like receptor 4 (TLR-4), α5β1 integrin, and UEA-1, have shown limitations in clinical translation due to the species-dependent variable efficacy [33,40]. Intestinal immunization is a series of cellular processes starting at the induction site, which is M cells in FAE of PP. Subsequent immune reactions require exposing the antigen to dendritic cells by M cells’ transcytosis. In this regard, although M cells act as a portal of entry in intestinal immunity, simply delivering the vaccines to the M cell cannot guarantee a potent oral immunity [40,41]. Therefore, translocation of antigens through M cells to subcellular compartmental APC cells is believed to be a key trigger of the process of inducing intestinal immunity. To achieve the most effective oral vaccine, a strategy to overcome these barriers should be considered in the design of the delivery system.

### 3.2. Immunological Barrier

In addition to overcoming physiological barriers, the success of oral vaccine also relies on the maintenance of both efficacy and safety as well as the prevention of oral tolerance. Oral poliovirus vaccine (OPV) has demonstrated its superior efficacy in eliciting both humoral and mucosal immunity [46]. However, the recent transition of OPV to parenteral inactivated poliovirus vaccine (IPV) due to the association with paralytic disease in rare cases exemplified the importance of a safety issue in the development of an oral vaccine. Oral tolerance is immunological unresponsiveness that arises after oral administration of an antigen and a potential problem in the development of an oral vaccine delivery system (Figure 2) [14,15]. Since early observations about the suppression of the T cell response after oral administration of ovalbumin or dinitrochlorobenzene, various subsequent experiments have also reported the induction of oral tolerance after the administration of soluble protein antigens, contact-sensitizing agents, heterologous blood cells, and inactivated viruses [16]. Although understanding the mechanisms of oral tolerance is still an ongoing issue, oral tolerance could be a natural outcome from the handling of a high total antigenic burden (i.e., hygiene) by immune cells. Several studies in animal models have suggested that oral tolerance appears in the activation-induced cell death (deletion), anergy, and most recently the induction of regulatory T cells [7,34]. Induction of regulatory T cells after mucosal delivery of antigens has been reported for more than 25 years, and recent studies indicated that four main types of regulatory T cells: (1) antigen-induced CD4^+^ T_H_2 cells [47], (2) CD4^+^CD45RB^low^ Tr1 [48], (3) CD4^+^ or CD8^+^ T cells producing TGF-β (T_H_3 cells) [49], and (4) CD4^+^CD25^+^ regulatory T cells (T_reg_ cells) [50,51] may induce or expand antigen-mediated oral tolerance [7]. Anergy and deletion of specific T cells have been reported after administration of either large quantity of soluble proteins or massive antigen doses [34,52,53]. Current approaches to avoid oral tolerance have been rooted in escaping anergy or deletion of T cells by the application of controlled release technologies and the development of better adjuvants.

## 4. Current Oral Vaccine Delivery Systems

### 4.1. Delivery Strategies of Oral Vaccines

**Figure 3:**
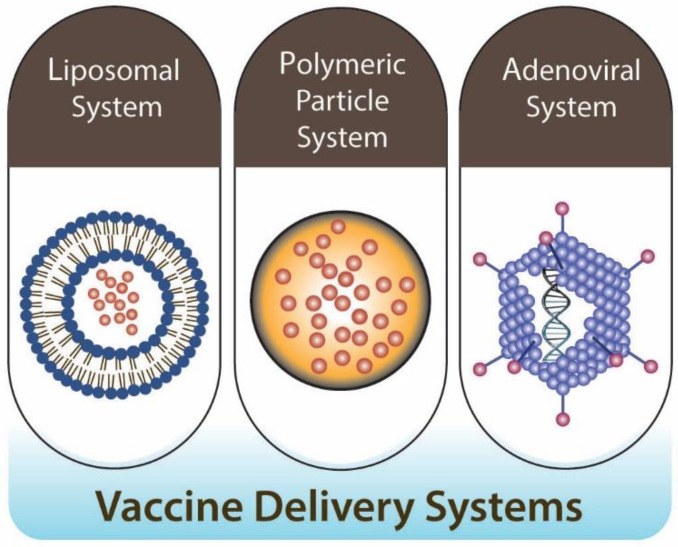
Oral vaccine delivery systems. Design and application of appropriate antigen-delivery vehicles for oral vaccination have been focused on using three different types of delivery systems: lipid-based (e.g., liposomal system), particle-based (e.g., polymeric particle system), and adenoviral vectors (e.g., adenoviral system). Each of these delivery systems has distinctive roles in the delivery of the oral vaccine.

Historically, oral vaccine delivery aimed at inducing intestinal immunity through the gut-associated mucosal tissues. However, mucosal sites are highly compartmentalized, and not all sites have the equivalent potential to elicit immunity against antigens in vaccines [54]. To deliver vaccines through the oral route, vaccines have to overcome significant challenges, including the acidic pH (especially stomach), poor absorption properties of epithelium cells in the GI tract, and generally poor immunogenicity [5,55,56,57,58]. To overcome these challenges, several delivery technologies applicable to oral vaccination are currently progressing in preclinical and clinical settings [59,60]. Current applicable oral vaccine delivery systems are broadly categorized into the liposomal system, polymeric particles, and the adenoviral vector system, and with or without live attenuated vaccines application, which can be useful to increase vaccine potency via in vivo proliferation (Figure 3).

### 4.2. Liposomal System

Liposomes and their derivatives are hollow spherical vesicles having one or more lipid bilayer. The liposomes can absorb hydrophilic DNA, RNA, antigens in the water-filled core, and hydrophobic drugs will move to the lipid bilayer. The liposomal delivery system provides several advantages, such as low immunogenicity, high biocompatibility, and antigen/immunomodulator protection [58]. As an oral vaccine delivery system, the liposome system was modified with a specific polymer or receptor to improve targeting efficiency and stability. Recently, Ma et al. [41,61] reported PLGA (poly (d,l)-lactide-co-glycolide), a polymerized lipid nanoparticle, can protect antigens in vaccines from degradation in the harsh GI environment and induce both mucosal and systemic antigen-specific immune responses. However, there remained critical problems, such as low GALT transportation and poor absorption of intestinal epithelial cells for oral liposomal vaccine delivery. These obstacles induce the immunological hypo-responsiveness and requirement of a relatively large dose of oral vaccine [41]. To solve these problems, several approaches have been tried, such as surface modifications to have physical or chemical properties that enhance the interactions between the liposome and the endocytosis pathways of the gut [62,63]. Interestingly, M cells have better liposome-transporting properties and effective interactions with immune cells compared to other intestinal epithelial cells [64,65]. The development of an oral vaccine carrier with lipid nanoparticles (NPs) conjugated with *Ulex europaeus* agglutinin-1 (UEA-1) demonstrated enhancement of the transport efficiency of lipid NPs across the intestinal mucosa through M cells and the induction of phagocytosis [41]. However, their efficiency at inducing an immune reaction remained relatively low compared to the intramuscular administration of vaccines. As an alternative, Jain [66] and Wang [67] added mannan moieties as a specific ligand to target the mannosyl receptors of antigen presenting cells (APCs) including macrophages, B cells, and dendritic cells. This approach elicited a better immune-response by the generation of both mucosal IgA and serum IgG, compared to intramuscular injection. The lipid-based vaccine delivery systems have demonstrated their usefulness for dozens of years, with several preclinical and clinical trials. However, insufficient immunization effect still posed a major problem in oral delivery. Therefore, the optimal oral vaccine delivery with liposomal carriers requires several additional features such as antigen stabilizer, liposomal carriers, protectors from environmental harm, and enhancers to increase absorption of vaccines by M cells.

### 4.3. Polymeric Particle System

Biodegradable and biocompatible polymers are useful vehicles for the drug, gene, metal, and nanoparticle delivery. In vaccination, they play a role in improving the targeting ability of vaccines to specific organs and the environment by releasing antigens from their highly tunable and dynamic structural transitions (e.g., site-specific degradation) in response to biological stimuli [68,69,70,71]. Several fabricated polymers, such as PLGA, PLA, PEG, and dendrimers, have been used as an effective platform for vaccine delivery due to their great stability and biocompatibility, as well as high loading capacity [72,73,74,75]. However, these polymers posed several critical limitations, including dramatic digestion by the abundant enzymes (e.g., esterase, trypsin, proteases) in the GI tract, poor absorption by the follicle-associated epithelium, and high dropout rates for the booster doses [76,77]. Therefore, they were defeating the purpose of mass immunization.

To overcome these limitations, natural polymers, such as β-glucan, chitosan, starch, and hyaluronic acid have been used as an alternative material for oral vaccine delivery instead of fabricated polymers [78,79,80]. For example, starch is considered an attractive candidate for an oral delivery system of biomolecules due to its abundance in nature and prior approval for many alimentary and pharmaceutical uses [81]. Alginates are natural polysaccharides extracted from brown seaweed. Their characteristic feature is the capability to form hydrogels in the presence of divalent cations. Alginate vehicles were effective for mucosal vaccination in animal species [82,83]. However, the use of alginate vehicles for oral vaccine delivery has been reported to cause a burst release of antigens due to the macroporous structure, which is the place for antigen absorption of the particles. This structural problem requires strategic approaches to prevent desorption or the degradation of the antigens in the GI environment [79]. Chitosan has been extensively studied for the delivery of therapeutic proteins and antigens, mainly via mucosal routes because of its excellent mucoadhesive and absorption-enhancing properties for M cells of the follicle-associated epithelium (FAE) [78,84,85,86]. Various studies have demonstrated the activation of the dendritic cells, macrophages, and lymphocytes by the chitosan-mediated oral vaccine delivery system. The formulation of chitosan-based nanoparticles has been developed by either chemical modification or complexation with oppositely charged molecules through electrostatic interactions [86,87]. With its nontoxicity, biodegradability, biocompatibility, and bio-adhesion ability, current chitosan applications still faced several challenges, such as water insolubility at physiological pH and easy degradation in acidic media, such as the GI tract [79,86,88,89,90]. Beta-glucans are one of the best candidates for oral vaccine delivery, which can provide target delivery to APCs and generally recognized as safe (GRAS). Particulate delivery systems with β-glucan allowed for efficient trapping of the antigen in the vehicle while retaining its conformations and integrity and demonstrating their targeting of APCs across the M cells in Peyer’s patches and strong potency to elicit durable immune responses [80,91,92,93,94]. These promising antigen carriers are derived from the cell wall of *Saccharomyces cerevisiae* (baker’s yeast) and are mainly composed of β-1,3-d-glucans, a ‘microbe-associated molecular patterns’ (MAMPs) with adjuvant ability. De Smet et al. and Baert et al. have tried to use β-glucan for antigen delivery vehicles. Their formulation with β-glucan has successfully promoted the maturation of dendritic cells and increased systemic antibody responses with expected oral immunization [80,91]. These polymer-based delivery systems have attractive properties that solve the critical problems for oral vaccination. However, their applications require compulsory evaluation of humoral response before application to clinical trials.

### 4.4. Adenoviral System

Adenoviruses are well known for the ability to induce both strong antibody and T cell responses to the transgenic antigen. As an oral vaccine delivery approach, viral vector-based vaccines can circumvent pre-existing immune responses against adenovirus and generate substantial transgene-specific immune responses. However, the enteric coating process is required to protect the vaccine because adenovirus cannot protect against the degradation of the vaccine in the stomach. For controlled and targeted antigen delivery, there have been several approaches such as polymerization and the tablet system. Licensed oral adenovirus vaccines with serotypes 4 and 7 provide a model for the use of live recombinant adenoviruses in oral immunization. Live oral adenoviruses have been used to prevent acute respiratory disease caused by adenoviruses 4 and 7 [95]. These vaccines contain a lyophilized live and wild-type virus incorporated into enteric tablets that protect against the acidic pH in the stomach. After oral administration, the live virus was released into the intestine where asymptomatic replication occurs. That vaccine generated an immune response that was over 95% effective in preventing adenovirus 4 and 7 induced respiratory illness in a clinical trial [96]. The recombinant adenovirus tablet provided genome stability and elicited vigorous humoral and cellular immune responses including the GI tract [97,98]. However, current licensed adenoviral systems must be coated with the enteric tablets to overcome their degradations in the acidic pH in the stomach after oral administration [96]. oral adenovirus application required other systemic application to improve the immunization with an increase in the oral uptake property and specific cell target, such as M-cells.

## 5. Direction to Enhance Oral Vaccination 

### 5.1. Biological Target-M Cell 

M cells are key players in particle transport from the intestinal lumen to the GALT [33]. Therefore, M cells are extremely desirable targets for oral vaccine delivery [42,44,99]. For efficient M-cell targeting, the first approach can be the actual enhancement of either total number or function of M cells. Following bacterial exposure, several studies suggest that the proportion of M cells in the FAE can be increased rapidly. For example, M cell numbers were increased two-fold after exposure of germ-free mice to *Salmonella typhimurium* [100]. In addition, Gebert et al. demonstrated that short-term exposure to *Streptococcus pneumonia* R36A leads to an increased transport capacity of M cells [101]. The bacterial exposure provided clues about either increasing the number of M cells or boosting their transport capacity. Along with these benefits, bacterial exposure itself posed potential side effects, such as the increased probability of inducing food allergies and inflammatory diseases [2]. A recent study reported a bacteria-free M cell boosting system after pre-systemic administration of the receptor activator of the NF-κB ligand (RANKL) before delivery of the oral vaccine [102]. This system demonstrated the induction of mucosal and humoral immune responses after delivery of the oral vaccine to the M cells without the risks associated with bacterial exposure.

Efforts to generate ligands to label M cells provided clues for M cell targeting. In 1999, some excitement surrounded a discovery of sialylated Lewis A antigen (SLAA) selectively expressed on M cells from biopsies of human PP [103]. In subsequent studies, researchers found that M cells express a specific carbohydrate moiety (α-L-fucose) on the apical surface [104,105]. Lectin subtypes, such as *Ulex europaeus* agglutinin 1 (UEA-1) and *Aleuria aurantia*, have shown their high specificity for α-L-fucose on M cells [104,106]. Based on these observations, there have been intensive efforts for oral vaccine delivery to M cells by conjugating lectin to liposomes [99], latex particles [107], or poly (d,l-lactide-coglycolide) (PLG) particles [106]. Oral administration of these particles results in significant induction of intestinal immunity indicated by substantial increases in sIgA, Th1-cytokine IL-2, and IFN-γ [99,106,107]. Although lectin’s potential to target M cells is highly applicable to improving intestinal immune response, UEA-1 lectin has limited value in vaccine delivery because lectin is toxic, easily degraded in the intestine, and inherently immunogenic [23]. Moreover, it is unfortunate that there is no expression of lectin receptors in human PP or murine M cells [103,104]. While stable and nontoxic, lectin mimetic small molecules have potential in oral vaccine delivery, but the existence of the corresponding receptor in human M cells is a prerequisite.

The use of antibodies has also been tried for M cell-specific targeting. For example, an oral application of tetanus toxoid (TT) or botulinum toxoid (BT) conjugated with M cell-specific monoclonal antibody (mAb NKM16-2-4), together with adjuvant, induced high levels of antigen-specific mucosal IgA, as well as serum IgG [108]. Shima et al. [109] also reported oral administration of biotinylated ovalbumin peptide (bOVA) conjugated with anti-GP-2-Streptavidin (SA)-induced active mucosal immunity against OVA in mice by targeting the conjugate to the glycoprotein 2 (GP2), a protein specifically expressed on M cells. Interestingly, recent studies reported the utilization of a mucosal sIgA antibody complex for M cell targeting, which is based on the discovery of Dectin 1, a novel IgA receptor in M cells for transportation of sIgA from luminal secretions into GALT [110,111]. Oral delivery of sIgA complex (p24-sIgA), conjugated with p24HIV antigen combined with *E. coli* heat-labile enterotoxin (HLT) as an adjuvant, elicited both humoral and cellular immune responses against p24 at the systemic and mucosal level [111].

The understanding of M-cell ontogeny has led to the establishment of an in vitro M-cell model using a cell culture system and providing a way to perform gene expression studies with M cell-like cells to discover M cell specific receptors [112]. The in vitro M-cell model is based on the phenotype conversion of Caco-2 cells into M cell-like cells with the assistance of B lymphocytes [112]. The M cell-like cells are generated either in a co-culture system by intercalation of B cells within Caco-2 monolayer or a separate compartment system without physical contact of B cells with the monolayer [113]. A gene expression study combined with PCR and in situ hybridization demonstrated that a range of genes was selectively upregulated in the M cell-like cells generated from the co-culture system. These included claudin 4, laminin β3, tetraspan TM4SF3, and matrix metalloproteinase (MMP 15) [114]. Of these, comparisons between these genes and the human PP gene profile revealed several conserved genes, such as claudin 4 and TM4SF3. Claudin 4, in particular, appears to be a protein having a dual location at tight junctions between M cells and enterocytes, as well as in the cytoplasm as a receptor in both M cells and enterocytes. Therefore, it could have application potential for an M cell-specific oral vaccine delivery system using surface-conjugated peptides having high affinity to claudin 4 to induce intestinal immunity [115].

Phage display is a promising method to identify peptide sequences interacting with immobilized proteins, carbohydrates, and peptides displayed by cultured cells without knowledge of their molecular properties [116]. Application of phage display can provide peptide sequences interacting with unknown ligands expressed on the surface of M-like cells. For example, Fievez et al. [117] reported the identification of novel lead peptides targeting human M cells from an in vitro human M-cell model. T7 phage display library screening provided two peptide sequences (CTGKSC and LRVG) specific to M-like cells. PLGA polymeric nanoparticles grafted with these selected peptides demonstrated a significant increase in their transport across the M-cell model by eight and four times, respectively, when compared to non-grafted nanoparticles. Other researchers also reported the identification of an M cell-homing peptide by phage display, from which the CKSTHPLSC (CKS9) peptide sequence was selected for grafting with chitosan nanoparticles (CNs) [118]. CKS9 peptide prominently enhanced the targeting and transcytosis ability of CNs to M cells of PP. Moreover, oral delivery of membrane protein B of *Brachyspira hyodysenteriae* (BmpB) loaded into CKS9-WSC (water-soluble chitosan)-PLGA microparticles demonstrated the induction of sIgA and systemic IgG in mice [78].

### 5.2. Self-Adjuvants

Current challenges in the development of vaccines are imprecise antigen display, and the use of heterogeneous adjuvants whose mechanisms of action are extremely complex and incompletely understood [119]. Synthetic peptides are particularly useful as subunit antigens because of a precise definition of epitopes against an immune response. While subunit antigens are a valuable tool for overcoming current antigen issues in vaccine delivery, their poor immunogenicity requires co-administration with strong adjuvants [119]. Many adjuvants currently employed or under development have intrinsic problems in their definition, formulation, purification, and characterization due to complex molecular constituents or heterogeneous mixtures [120]. For example, current adjuvants are particulates such as aluminum salts, oil emulsions, toll-like receptor (TLR) ligands, immunostimulating complexes (ISCOMs), and other biologically-sourced materials, such as cholera toxin subunit B (CTB) or *E. coli* heat-labile enterotoxin (LT) [121]. While they have shown good immunostimulating effects, these adjuvants are not easily adjusted independently within a given vaccine formulation, making for difficult optimization and mechanistic study. As a potential solution associated with adjuvants in vaccine delivery, self-adjuvanting or adjuvant-free systems are suggested for next-generation vaccine delivery systems. For example, Rudra et al. [121] reported a potential adjuvant-free system from the development of epitope-bearing-self-assembling peptides by linking ovalbumin sequences to nanofibers using a short C-terminal peptide extension. These peptides self-assembled into fibrils that induced the expression of IgG1, 2, and 3 against epitopes on the fibrils in a level similar to the complete Freund’s adjuvant (CFA). In addition, oral delivery of the self-adjuvanting peptide-nanofiber-CaCO_3_ composite microparticles (OVA-KFE8/CaCO_3_) demonstrated potential as adjuvant-free oral vaccine delivery vehicles for the induction of a mucosal antibody response [122]. Along with these self-adjuvanting systems, Zaman M. et al. [123] also demonstrated a self-adjuvanting mucosal vaccine effect against group A streptococcus by the development of a hybrid system from conjugating both B and T cell epitopes to lipopeptide, a Toll-like receptor 2 targeting lipid moiety. Therefore, the self-adjuvanting system has potential in the preparation of safe and reliable next-generation oral delivery vaccine systems.

### 5.3. Delivery System-Hybrid Delivery System

Current licensed oral vaccines, including polio, cholera, rotavirus, and influenza are predominantly live-attenuated formulations, which closely mimics natural infections and effectively elicits strong intestinal immunity [7]. However, risks include inflammation, uncontrolled replication, and reversion potential to pathogenic disease [23]. Subunit vaccines have gained attention as a safe alternative due to the use of only part of the target pathogen provoking a response from the immune system or a gene construct encoding only for viral immunogens [23,33]. Although they have superior safety compared to conventional vaccines, current concerns are low immunogenicity and limitations in DNA carrying capacity [124]. Current challenges to these issues are the use of live, food-grade, noninvasive, and nonpathogenic bacteria as antigen vehicles [124,125,126,127,128]. For example, Zhang et al. reported the development of an oral cancer vaccine by delivering carcinoembryonic antigen (CEA) with *Lactococcus lactis*, a food-grade strain of lactic acid bacteria (LAB) [124]. Oral administration of this vaccine demonstrated the induction of sIgA against CEA without causing oral tolerance. Another live recombinant LAB system also demonstrated the enhancement of intestinal immunity by delivering antigen and recombinant adjuvant, IL-6 combined with M cell targeting peptide (CK9) [128]. M cell-specific targeting increased bioavailability, as well as induction of both Th1- and Th2-type immune responses, simultaneously, and without oral tolerance. Therefore, future oral vaccine delivery systems may stem from using a hybrid delivery system, such as a live recombinant vaccine delivery system combined with a natural food-grade bacterial strain.

## 6. Conclusions and Prospects

Successful oral vaccine delivery requires an understanding of the biological basis of the intestinal immune system and delivery criteria. The knowledge of molecular anatomy and the ontogeny of the intestine and its cellular components helps to capture the mechanistic view of the interaction of pathogens or particles with the surface of intestinal M cells, absorption, and even association with the induction of intestinal immunity. Furthermore, the development of the in vitro human M-cell model paved the way for the discovery of human M cell targets, as well as overcoming intra-species variations in M-cell properties, which have been major hurdles in the application of oral vaccine delivery systems in humans. The development of potent oral vaccine delivery systems needs to address several challenges, including maintenance of stability in the GI environment, enhancement of bioavailability of vaccines by targeting M cells, and the induction of strong, balanced immune responses without inducing tolerance through the regulation of the M cell-mediated intestinal immune induction system. Conventional particle delivery systems with liposomes, polymers, and adenoviruses have made significant contributions to oral vaccine delivery by achieving the maintenance of stability and improvement of delivery efficiency to the intestine. However, it is still a challenge to achieve M cell-specific targeting and strong and balanced intestinal immune induction.

Recent gene expression and phage display studies with the in vitro human M cell model led to the identification of potential human M-cell targets. Based on these target identifications, using conventional liposomal, polymeric, or adenoviral vehicles, or monoclonal antibody targeting strategies, it might be possible to better direct oral vaccines specifically to human M cells. However, it is still problematic to differentiate human M cells from neighboring enterocytes due to the possible expression (albeit at lower levels) of the same targets on the cell surface. The understanding of human M cell function, identification of more specific surface markers, and the improvement of intestinal M cell-like models are crucial for the design and development of M cell-targeted vaccines. An adjuvant is a requirement for strong induction of intestinal immunity in currently available oral vaccine delivery systems. Cholera toxin (CT) and heat-labile enterotoxin (LT) are two of the most promising mucosal adjuvants derived from bacteria, but they are limited by the potential safety issues associated with the use of native toxin. Several types of research reported promising results using the self-adjuvanting system combined with defined antigen subunits as an adjuvant-free oral vaccine delivery system. Furthermore, hybrid oral vaccine delivery systems using natural food grade bacteria as a vehicle for vaccines have also demonstrated implications for the future direction of oral vaccine delivery.

Vaccine technology has shown continuous evolution since its inception. Advances in related areas, such as genetic and metabolic engineering, have allowed for attempts to mimic pathogen entry routes into M cells and possibly other routes of antigen uptake. These works could assist in the development of ligand-mediated targeting of particulate vaccine vehicles. In summary, the progress of oral vaccine delivery has required answering many questions from the biological basis and delivery criteria. However, with increased understanding of intestinal biology and oral immunity, as well as the development of various novel delivery systems and adjuvants, there is greater hope and increased applications into therapeutics, such as cancer immunotherapy, for further development of improved oral vaccines.

## Figures and Tables

**Table 1:** Development of oral and nasal vaccines.

	Mode of treatment	Name	Desease	Company	Clinical phase	Ref.
Nasal Vaccines	Current treatment (including Licenses)	FluMist^®^ Quadrivalent	Influenza A subtype and type B viruses	MedImmune, Gaithersburg, MD, USA	Out in Market	[11,17,18,19]
		Nasovac-S^TM^	Influenza A (H1N1)	Cipla, Mumbai, India		[10,17]
		Nasalflu	Influenza A (H1N1 and H3N2) and type B	Crucell, Leiden, The Netherlands		[20]
	Pre-clinical or clinical trial	μco^TM^	Anti-emetic migraine, flu	SNBL, Tokyo, Japan	Phase II, Phase I, pre-clinical	[6]
		Optinose	Chronic Rhinosinusitis (CRS), Chronic Sinusitis (CS)	OptiNose, Yardley, PA, USA	Clinical trials (various)	[6]
		Flumis Fleuenz	Flu	MedImmune, Gaithersburg, MD, USA	FDA & EMA	[6]
		ChiSys	Avian influenza virus (H5 and H7 subtypes)	Archimedes Pharma, Reading, UK	Phase I, pre-clinical	[6,21]
Oral Vaccines	Current treatment (including Licenses)	Dukoral^®^	Vibrio Cholera	VALNEVA, Lyon, France	Out in Market	[22,23,24]
		Biopolio^TM^ B1/3	Types 1, 2 and 3 attenuated poliomyelitis viruses (Sabin Strains)	Bharat Biotech, Telagana, India		[22,25]
		Rotarix^®^	Rotavirus	GSK, Brentford, UK		[19,22,26]
		RotaTeq^®^	Rotavirus	MSD, Kenilworth, NJ, USA		[19,22,26]
		Vivotif^®^	Salmonella typhi	PaxVax, Redwood city, CA, USA		[19,22,23]
		Anflu^®^	3 influenza virus strains (H1N1 influenza A virus subtype, H3N2 influenza A virus subtype, influenza B)	Alco Pharma, Dhaka, Bangladesh		[27]
		Euvichol^®^	Vibrio Cholera	Eubiologics, Seoul, South Korea		[28]
		Cholvax^®^	Vibrio Cholera	Incepta, Dhaka, Bangladesh		[28,29]
		Shanchol^®^	Vibrio Cholerae	SANOFI, Paris, Frnace		[19,24,29]
	Pre-clinical or clinical trial	LATTE-2	Hepatitis C virus	GSK, Brentford, UK	Phase IIb	[30,31]
		CholeraGarde^®^	Human immunodeficiency virus	AVANT Immunotherapeutics, Needham, MA, USA	Phase II	[24,32]
		RV3-BB	Rotavirus	GSK, Brentford, UK	Phase II, Phase I	[26]
		ORV 116E	Rotavirus	SAS, Delhi, India	Phase III, Phase II, Phase I	[26]
